# Deep Reinforcement Learning-Based Resource Management in Maritime Communication Systems

**DOI:** 10.3390/s24072247

**Published:** 2024-03-31

**Authors:** Xi Yao, Yingdong Hu, Yicheng Xu, Ruifeng Gao

**Affiliations:** 1 School of Information Science and Technology, Nantong University, Nantong 226019, China; 2110310028@stmail.ntu.edu.cn (X.Y.); huyd@ntu.edu.cn (Y.H.); yc.x@ntu.edu.cn (Y.X.); 2 School of Transportation and Civil Engineering, Nantong University, Nantong 226019, China

**Keywords:** deep reinforcement learning, beam allocation scheme, deep Q-network

## Abstract

With the growing maritime economy, ensuring the quality of communication for maritime users has become imperative. The maritime communication system based on nearshore base stations enhances the communication rate of maritime users through dynamic resource allocation. A virtual queue-based deep reinforcement learning beam allocation scheme is proposed in this paper, aiming to maximize the communication rate. More particularly, to reduce the complexity of resource management, we employ a grid-based method to discretize the maritime environment. For the combinatorial optimization problem of grid and beam allocation under unknown channel state information, we model it as a sequential decision process of resource allocation. The nearshore base station is modeled as a learning agent, continuously interacting with the environment to optimize beam allocation schemes using deep reinforcement learning techniques. Furthermore, we guarantee that grids with poor channel state information can be serviced through the virtual queue method. Finally, the simulation results provided show that our proposed beam allocation scheme is beneficial in terms of increasing the communication rate.

## 1. Introduction

The sixth-generation (6G) wireless communication aims to expand network coverage and improve network performance [1,2]. Maritime communication, as an important component of wireless communication, has received increasing attention with the growing maritime economy.

In general, maritime communication is composed of satellite communication and communication based on nearshore base stations (BSs). Satellite communication systems, such as the Global Maritime Distress and Safety System (GMDSS), Iridium system, and International Maritime Satellite System (Inmarsat) [3,4,5] can cover the large maritime environment, meeting the communication requirements of maritime users. However, the high cost and latency of satellite communication are the main challenges faced in maritime satellite communication [6]. Communication systems based on nearshore BSs can be integrated with terrestrial communication systems, which effectively reduces cost and latency. Nevertheless, compared to terrestrial communication, maritime communication is subject to multiple factors. The refractive index fluctuation caused by the uneven atmospheric pressure and temperature, namely turbulence, reduces the performance of the communication system [7], and, due to the lack of scatterers in the vast maritime environment, the scattering of electromagnetic waves affects communication performance [8]. Furthermore, maritime communication users exhibit locally dense, and overall sparse, distribution characteristics. These factors make it inappropriate to directly address the communication requirements of maritime users through traditional terrestrial communication.

Currently, there are some nearshore communication systems and networks designed for maritime communication. The Long Term Evolution (LTE)–Maritime project aims to meet the communication requirements of maritime users using ground infrastructure [9]; it can support a high data rate while providing coverage around 100 km from BSs. The mesh TRITON network, based on IEEE 802.16, focuses on dense wireless mesh networks in maritime nearshore areas for maritime users [10]. The nearshore communication systems, such as the navigation telex (NAVTEX) system and the automatic identification system (AIS) provide services for information broadcasting, voice, and ship identification [11]. However, the above communication systems and networks are merely direct applications of terrestrial communication systems in maritime environments. At present, there is still a lack of communication schemes based on the characteristics of the maritime environment and maritime users. To solve the above problems, beamforming technology can provide directional coverage, ensuring communication quality for users within the coverage area [12,13].

In maritime communication systems, beamforming technology can be used to solve the issue of communication distance and improve user communication quality [14,15]; it can further enhance communication system performance through its combined application with non-orthogonal multiple access (NOMA) technologies [16]. However, further research is needed on how to manage beam resources, and when and where to make beam management decisions. On the one hand, maritime communication faces challenges such as long distances between BSs and users, significant transmission delays, and high interference [17]. It is difficult to obtain the CSI in the maritime environment [18], and the traditional communication resource allocation schemes based on CSI are difficult to employ in the maritime environment [19]. On the other hand, there is still a lack of relevant research on the distribution characteristics of communication users in maritime environments. So, it is necessary to design an efficient beam management scheme to cater to the characteristics of the maritime environment and users.

The essence of a beam management scheme is essentially a combinatorial optimization problem; its computational complexity increases continuously with the growth of the dimension of combinatorial optimization states. Traditional methods are not applicable when the state space is large. Reinforcement learning (RL) can solve this problem by structuring combinatorial optimization as a sequential decision process. It continuously interacts with the environment and updates iterations based on real-time data, optimizing the choices made. RL has been widely applied in the scenario of resource allocation in maritime environments [20,21]. In a rapidly changing vehicular environment, Liang et al. [22] effectively improved the transmission rate in the end-to-end communication link. It has been shown that multi-agent RL can achieve significant performance gains by allocating appropriate resources in the face of uncertain environments [23].

Nevertheless, Shi et al. [24] also point out that, as the number of serviced users increases, which corresponds to the growth in the state space of RL, there is a higher requirement for online devices, which will reduce the efficiency of service devices. Meanwhile, due to the dynamic nature of the maritime environment, it is challenging to fully describe the state information of all communication users using a Q-network in RL. Hence, we consider using the method of deep reinforcement learning (DRL) to address the resource allocation problem. DRL as an effective method has achieved great performance in resource management [25,26]. In [27], Hu et al. propose a DRL framework model to address the decision problem of dynamic resource allocation in satellite communication. In [28], Qian et al. effectively reduced the total energy consumption of the entire maritime Internet of Things network by utilizing relay devices for resource allocation through a DRL framework. All of these papers demonstrate the effectiveness of using a DRL method for resource allocation in communication networks. Furthermore, in order to further reduce the complexity of communication systems, an optimized algorithm using a grid-based method to tackle the resource allocation management problem is proposed in [29]. Similarly, we can divide the sea area covered by the BS’s transmitted signal into multiple grids.

In this paper, a virtual queue-based deep reinforcement learning (VQDRL) beam allocation scheme for maritime communication systems is proposed, wherein the CSI is unknown, aiming to maximize the communication rate of the maritime communication system. Due to the sparse features of maritime users, an optimized algorithm using a grid-based method is employed to tackle the resource allocation management problem. After modeling the maritime communication system and VQDRL framework, we employ neural networks to allocate beam resources. Furthermore, the neural network is trained based on the communication rate of maritime users to obtain the optimal beam allocation scheme. This paper has three main contributions which are as follows:

1. Due to the complexity of the maritime environment, a grid-based method is adopted to discretize the coverage area, reducing the complexity of resource management.
2. A VQDRL resources allocation scheme is employed in grids with unknown CSI. By continuously training the neural network to optimize its output, we obtain the most effective beam allocation scheme.
3. A virtual queue method is employed in maritime communication, it can ensure that grids with poor channel states can be served.

The remainder of this paper is organized as follows. Section 2 introduces the system model. A VQDRL resources allocation scheme is proposed in Section 3, and the simulation results are given in Section 4. Finally, we conclude this paper in Section 5.

## 2. System Model and Problem Formulation

### 2.1. System Model

**Figure 1:**
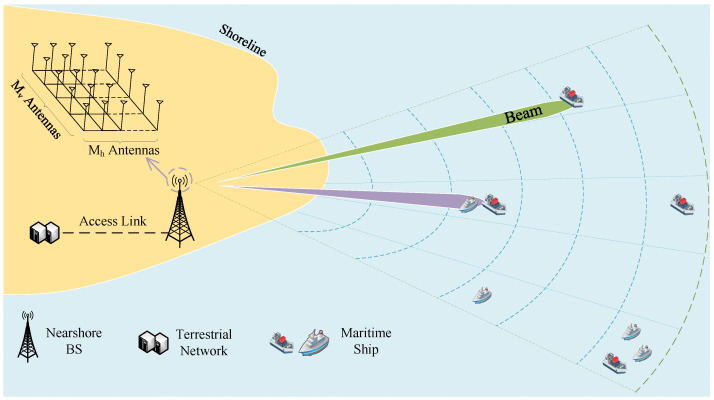
Maritimecommunication system model.

The maritime communication system model is shown in Figure 1. A uniform antenna array with a total of $N_{t}=N_{t}^{h}\times N_{t}^{v}$ antennas is deployed on the base station (BS). There are $N_{t}^{h}$ antennas in the horizontal direction and $N_{t}^{v}$ antennas in the vertical direction. Based on the characteristics of the antennas, a total of *N* beams are generated. After dividing the maritime environment using the grid-based method, we can get *M* grids. Now, $N=\left\{1,\ldots n,\ldots N\right\}$ and $M=\left\{1,\ldots m,\ldots M\right\}$ represent the set of beams and grids, respectively. Here, *n* and *m* denote the index of beams and grids. When we obtain the distance $d_{m}$ and horizontal angle $\theta_{m}$ from grid *m* to the BS, we can describe the grid information by using the set $s_{m}=\left\{d_{m},\theta_{m}\right\}$. And the received signal at the *m*-th grid with the *n*-th beam can be expressed as

(1) $$y_{m,n}=\sqrt{\frac{P_{t}G_{m}}{d_{m}^{\alpha }}}h_{m}w_{n}x_{m}+\sqrt{\frac{P_{t}G_{m}}{d_{m}^{\alpha }}}\sum_{\begin{array}{c} b\in N\\ b\neq n \end{array}}\sum_{\begin{array}{c} a\in M\\ a\neq m \end{array}}h_{m}w_{b}x_{a}+n_{0}$$

where $P_{t}$ represents the transmit beam power, $G_{m}$ denotes the antenna gain for grid *m*, α is the path loss coefficient, and $h_{m}$ and $w_{n}$ denote the channel from grid *m* to the BS and the unit-norm precoding vectors of beam *n*, respectively, which satisfy $\left|\left|w_{m}\right|\right|=\left|\left|w_{b}\right|\right|=1$. Let $x_{m}$ and $x_{a}$ denote the transmission signal from the BS to the grid *m* and *a*; moreover, the $n_{0}∼\mathrm{CN}\left(\begin{array}{c} 0,1 \end{array}\right)$ is the additive complex white Gaussian noise.

### 2.2. Problem Formulation

From Equation (1), when we transmit a signal from the BS to the grid *m* with beam *n*, we can get the received SINR of the users at time slot *t* using the following equation:

(2) $${\mathrm{SINR}}_{m,n}\left(t\right)=\frac{P_{t}G_{m}d_{m}^{-\alpha }{\left|h_{m}\left(t\right)w_{n}\left(t\right)\right|}^{2}}{1+P_{t}G_{m}d_{m}^{-\alpha }\sum_{\begin{array}{c} b\neq n \end{array}}^{b\in N}{\left|h_{m}\left(t\right)w_{b}\left(t\right)\right|}^{2}}$$

The communication rate can be expressed as

(3) $$R_{m,n}\left(t\right)=\log_{2}\left(1+{\mathrm{SINR}}_{m,n}\left(t\right)\right)$$

Consequently, the achievable communication sum-rate of the maritime communication system model is given by

(4) $$R_{\mathrm{sum}}\left(t\right)=\sum_{\begin{array}{c} m\in M \end{array}}\sum_{\begin{array}{c} n\in N \end{array}}R_{m,n}\left(t\right)$$

To maximize the accumulated communication throughput in a period set T={1,2,…,T}, the optimization problem can be expressed as

(5) $$\max\sum_{\begin{array}{c} t\in T \end{array}}\sum_{\begin{array}{c} m\in M \end{array}}\sum_{\begin{array}{c} n\in N \end{array}}R_{m,n}\left(t\right)K_{m,n}\left(t\right)$$

(5a) $$\begin{array}{c} s.t.\;K_{m,n}\left(t\right)(1-K_{m,n}\left(t\right))=0,\forall m\in M,n\in N,t\in T, \end{array}$$

(5b) $$\begin{array}{c} \sum_{\begin{array}{c} m\in M \end{array}}K_{m,n}(t)\leq P,\forall m\in M,n\in N,t\in T, \end{array}$$

(5c) $$\begin{array}{c} \sum_{\begin{array}{c} n\in N \end{array}}K_{m,n}(t)\leq P,\forall m\in M,n\in N,t\in T, \end{array}$$

(5d) $$\begin{array}{c} R_{m,n}\left(t\right)K_{m,n}\left(t\right)\geq R_{th}\left(t\right)K_{m,n}\left(t\right),\forall m\in M,n\in N,t\in T. \end{array}$$

where $R_{th}\left(t\right)$ denotes the minimum achievable communication rate for an *m* user in time slot *t*, and $K_{m,n}\left(t\right)$ represents whether the communication of beam *n* for grid *m* in the current time slot *t* was successful.

Constraints (5a) ensure that the value of $K_{m,n}\left(t\right)$ can be either 0 or 1. Constraints (5b) and (5c) denote that a maximum of *P* beams can be used to serve *P* grids at any given time slot. Constraint (5d) means that when the current communication rate is greater than the minimum rate or equal to the minimum rate, the value of $K_{m,n}\left(t\right)$ is 1, otherwise, the value of $K_{m,n}\left(t\right)$ is 0.

### 2.3. Deep Reinforcement Learning Model

**Figure 2:**
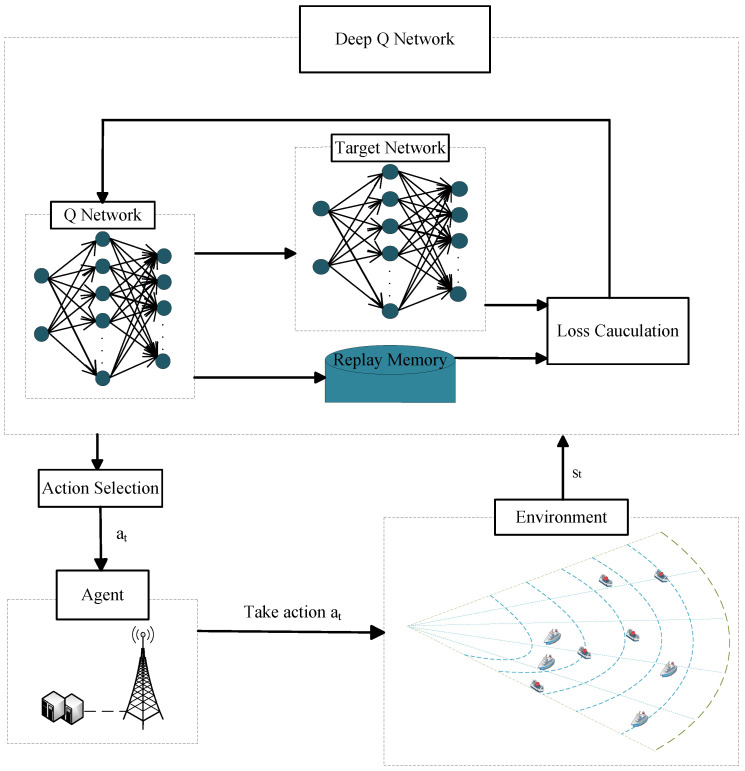
Deep reinforcement learning model.

For the optimization equation described above, it is impossible to obtain real-time channel state information $h_{m}\left(t\right)$ to adjust beam allocation schemes. The beam allocation problem is constructed as an RL system model and the RL algorithm can optimize the action-choosing behavior through massive interactions between the agent and environment. However, when the dimensions of the state space and action space are too large, traditional tabular-based RL algorithms face issues such as being time-consuming. The DRL addresses the limitations of traditional RL algorithms by using deep neural networks to select actions; it updates the weight parameters by minimizing the loss function to optimize the action-choosing behavior. The structure of the DRL model is shown in Figure 2.

#### 2.3.1. State Definition

In our model, we can obtain the state information of the grid that needs communication, namely as $s_{m}=\left\{d_{m},\theta_{m}\right\}$. The set of state information can be denoted as $S=\left\{s_{1},...s_{m},...s_{M}\right\}$. Moreover, the BS can handle communication requirements from *P* grids at any given communication time slot *t*, and the system state $s\left(t\right)\in R^{P\times 1}\subseteq S$.

#### 2.3.2. Action Definition

We define A(s(t)) as the set of available actions under state s(t). For any available action $a^{*}$, we have $a^{*}\in A\left(s\left(t\right)\right)$. Additionally, we use a(t) to denote the beam chosen scheme at time slot *t*.

#### 2.3.3. Reward Definition

We use reward r(t) to describe the degree of goodness or badness of taking an action a(t) in the current state s(t). In our model, we use r(t) to denote the condition of the communication rate. Typically, the reward value should be normalized in range $r\left(t\right)\in \left[0,1\right]$.

#### 2.3.4. Action Selection

To avoid the local optimum of DRL, the resource allocation decision is made by adopting the ϵ-greedy strategy, where the action is randomly selected with a probability of ϵ, while the action with largest action-values is selected with a probability of 1 − ϵ. We use Q(s(t),a(t);θ) to denote the chosen action through the Q-network. Action selection with the ϵ-greedy strategy can be expressed as:

(6) $$a\left(t\right)=\left\{\begin{array}{cc} \mathrm{random}, & \mathrm{with}\;\mathrm{probability}\;\epsilon \;\\ \underset{a^{*}}{\arg\max}Q\left(s\left(t\right),a^{*};\theta \right), & \mathrm{otherwise} \end{array}\right.$$

#### 2.3.5. Replay Memory

To alleviate the problems of related data and non-stationary distributions in our system model, a replay memory technique, which randomly samples previous transitions, and, thereby, smooths the training distribution over many past behaviors, is adopted. The experience item is stored in the form of quad-tuples (s(t),a(t),r(t),s(t+1)).

#### 2.3.6. Loss Calculation

In order to improve the deep neural network performance, we use $Q(\theta^{-})$ to denote the target network. During Q-network training, we update the weight parameters of the Q-network θ by minimizing the loss function, as given in Equation (3):

(7) $$L\left(\theta \right)=E[{\left(Q_{target}-Q\left(s\left(t\right),a\left(t\right);\theta \right)\right)}^{2}]$$

where the $Q_{target}$ is the target value defined below:

(8) $$Q_{target}=r\left(t\right)+\gamma \max_{a^{*}}Q\left(s\left(t+1\right),a^{*};\theta^{-}\right)$$

γ is the discount factor.

## 3. VQDRL Resources Allocation Scheme

In this section, a VQDRL resources allocation scheme is proposed to address the beam resource management problem in the maritime communication system. First, a virtual queue method is introduced to choose the grids of communication [30]. Then, the BS allocates the beam to the selected communication grids by using the proposed VQDRL resources allocation scheme, which can maximize the communication rate.

### 3.1. Virtual Queue Method

In communication, we can use DRL to select beam and grid pairs to maximize the communication rate. However, each grid has a different CSI, and directly using DRL to allocate beams may result in some grids with poorer channel conditions being unable to communicate. To address this issue, the method of virtual queue is introduced. By recording communication requirements and queue lengths in different grids, this method can ensure that communication is implemented in grids with poor channel conditions.

When we transmit signals to maritime users with communication requirements, we also need to allocate beam resources as frequently as possible to ensure the communication quality of those users with higher requirements. Meanwhile, we aim to send signals fairly to each maritime area with communication requirements. By recording the requirements of different grids, the virtual queue method can more frequently select grids with higher communication requirements for communication; it also ensures fairness in the communication among grids.

Let $r=[r_{1},\ldots ,r_{m},\ldots ,r_{M}]$ represents the vector of communication requirements of maritime users within each grid, and, for any grid *m*, we can calculate the proportion of a user’s requirement to the total grid requirements as follows:

(9) $$p_{m}=\frac{r_{m}}{\sum_{i=1}^{M}r_{i}}$$

Now, for each grid *m*, we create a virtual queue $V_{m}$ to denote the queue length at the beginning of the communication; it can be seen as the accumulated length of communication requirements up to the current communication time slot. The virtual queue length $V_{m}\left(t\right)$ is denoted according to the following dynamics:

(10) $$V_{m}\left(t\right)={\left[\begin{array}{c} V_{m}\left(t-1\right)+p_{m}-d_{m}\left(t-1\right) \end{array}\right]}^{+}$$

where ${\left[\begin{array}{c} x \end{array}\right]}^{+}≜\max\left\{x,0\right\}$, $d_{m}\left(t-1\right)$ denotes whether the communication rate in the current grid at time slot t−1 meets the communication rate requirement; it can be denoted as $d_{m}\left(t-1\right)$ = $\eta \sum_{n=1}^{N}K_{m,n}\left(t-1\right)$, and η is a coefficient to avoid excessively queue length.

At any given time slot *t*, the BS selects the grid to be served by beam resources and obtains the grid information $s_{m}$ using the following equation:

(11) $$s_{m}\in \underset{i\in M}{\arg\max}\left(V_{i}\left(t\right)\right)$$

### 3.2. VQDRL Resources Allocation Scheme

After obtaining the communication grids for the current time slot using the virtual queue method, we aim to maximize the communication rate by applying the DRL algorithm for beam allocation in these grids. However, the obtained grid information cannot be directly used as inputs for the neural network. In this case, the VQDRL resources allocation scheme is proposed to effectively utilize the obtained grid information and employ it as input to the neural network to obtain beam allocation schemes that maximize the communication rate.

According to Equation (11), when we obtain the transmitted grid information $s_{m}$ in the current time slot *t*, the VQDRL algorithm uses $s\left(t\right)=s_{m}=\left\{d_{m},\theta_{m}\right\}$ as an input to the neural network to obtain the output, which is the index of the allocated beam.

We can get different $Q(s\left(t\right),a^{*};\theta )$ values through the Q-network after inputting the state information s(t). In order to avoid the local optimum of VQDRL, we choose the action a(t) taken by the agent in the current state to allocate beam based on the probability of ϵ. When a randomly generated value is more than ϵ, we randomly select an action *a*, otherwise, we choose the action $\underset{a^{*}}{\arg\max}Q\left(s\left(t\right),a^{*};\theta \right)$.

After selecting the action a(t) for the current time slot, when the BS sends beams to the grids, the maritime users will get their communication rates $R_{m,n}\left(t\right)$. The reward r(t) is used to describe the condition of the communication rate, and we can obtain the next state s(t+1). It is obvious that when the constraint condition (5d) is satisfied under condition $\sum_{m,n}R_{m,n}\left(t\right)K_{m,n}\left(t\right)=\sum_{m,n}R_{th}\left(t\right)K_{m,n}\left(t\right)=0$, the reward r(t)=0. Otherwise, we define the function of reward r(t) as follows:

(12) $$r\left(t\right)=\left\{\begin{array}{cc} 1 & \mathrm{if}\;\sum_{m,n}R_{m,n}\left(t\right)K_{m,n}\left(t\right)\geq \sum_{m,n}R_{th}\left(t\right)K_{m,n}\left(t\right)\\ 0 & \mathrm{otherwise}\; \end{array}\right.$$

To further train our neural network while avoiding the issue of local optimization, we often use the replay memory D to store training data. Let us use the buffer size to describe the maximum amount of data stored in D and store the data set (s(t),a(t),r(t),s(t+1)) from the communication process into D. When the amount of data set stored is larger than the pre-defined batch size, we can randomly select a batch size of data from D to update the neural network. Otherwise, we continue the aforementioned communication process until the amount of data stored in D exceeds the batch size.

After selecting the data to be used for training, we update the weight parameters of the Q-network through the following process. We typically use the Bellman equation to update the Q-values like Equation (13) in RL:

(13) $$\begin{array}{c} Q\left(s\left(t\right),a\left(t\right)\right)\overset{\;}{\leftarrow }Q\left(s\left(t\right),a\left(t\right)\right)+\beta [r\left(t\right)+\gamma \max_{a^{*}\in A\left(s\left(t\right)\right)}Q(\\ s\left(t+1\right),a^{*})-Q\left(s\left(t\right),a\left(t\right)\right)] \end{array}$$

where β is the learning rate. Similar to RL, in DRL, based on the Bellman equation, we can obtain the weight update through gradient descent using the loss function. According to Equations (7) and (8), the gradient of loss function is calculated by calculating parameters as follows:

(14) $$\frac{dL(\theta )}{d\theta }=E\left[Q_{target}-Q\left(s\left(t\right),a\left(t\right);\theta \right)\frac{dQ(s(t),a(t);\theta )}{d\theta }\right].$$

In our model, we used the Adaptive Moment Estimation (Adam) optimizer to solve the gradient descent problem. It can adaptively adjust the learning rate to more effectively update the model’s weights. As a hyperparameter, the learning rate has a significant impact on weight updates for different values. In this model, we used a learning rate of $10^{-3}$ to train the model at the beginning of the training period and hyperparameter γ represents the discount factor.

After updating the weights of the Q-network, we determine whether it is time to update the weights of the target network for the current time slot. Typically, the target network parameter weights update every $N_{t}$ step. The parameter weights of the Q-network are assigned to the target network’s parameters, completing the update of the neural network.

## 4. Simulation Result

In this section, we simulated a maritime environment and deployed an antenna array at the BS, constructing beam resources firstly, then we employed the VQDRL algorithm to obtain the beam allocation schemes and observed the communication rate within the beam coverage area through simulation results, further demonstrating the effectiveness of our algorithm.

### 4.1. Simulation Enviroment Configuration

We deployed Uniform Planar Array (UPA) antenna arrays at the BS, and considered taking the Kronecker product of the Discrete Fourier Transform (DFT) codebook in the horizontal direction and vertical direction by using the Kronecker-product method. The 3D Kronecker-product-based codebook can select the appropriate beam to enhance the channel gain for the grid in both the horizontal and vertical direction [31]. It is generated as

(15) $$\begin{array}{c} C_{v}=[1,e^{\frac{j2\pi m}{\xi N_{v}}},\ldots ,e^{\frac{j2\pi (N_{t}^{v}-1)m}{\xi N_{v}}}{]}^{T}\\ C_{h}=[1,e^{\frac{j2\pi n}{N_{h}}},\ldots ,e^{\frac{j2\pi (N_{t}^{h}-1)n}{N_{h}}}{]}^{T}\\ C=C_{v}⊗C_{h} \end{array}$$

where **m** = 0, 1, *…*, $N_{v}-1$, **n** = 0, 1, *…*, $N_{h}-1$, $N_{v}$, and $N_{h}$ are the number of codewords in the vertical direction and horizontal direction, respectively, ⊗ denotes the Kronecker product, and ξ is a parameter to adjust the proportion which is determined by the maximum downtilt.

According to Equations (3) and (12), we need to simulate the maritime communication channel model to obtain the communication rate and the reward value to train the neural network. The maritime communication channel is assumed to follow the Rician distribution, expressed as follows:

(16) $$h_{m}=\sqrt{\frac{K}{K+1}}{\bar{h}}_{m}+\sqrt{\frac{1}{K+1}}{\hat{h}}_{m}$$

where *K* is the rice factor, $\hat{h_{m}}$ is the complex Gaussian random variables with zero mean and unit variance, which belong to a set of $C^{1\times N_{t}}$, and ${\bar{h}}_{m}$ is the channel mean vector. When we consider the antenna array arranged in a UPA, the channel mean vector of the *m*-th grid vector is expressed by [32,33]:

(17) $$\begin{array}{cc} {\bar{h}}_{m}= & [1,\ldots ,e^{j\frac{2\pi }{\lambda }d\left(n_{t}^{h}\sin\theta_{m}\sin\phi_{m}+n_{t}^{v}\cos\phi_{m}\right)},\\ & \ldots ,e^{j\frac{2\pi }{\lambda }d\left(N_{t}^{h}\sin\theta_{m}\sin\phi_{m}+N_{t}^{v}\cos\phi_{m}\right)}] \end{array}$$

where λ is the wavelength, *d* is the inter-antenna spacing, and $\theta_{m}$ is the horizontal angle of the BS to the *m*-th grid, while $\phi_{m}$ is the vertical angle of the BS to the *m*-th grid, which can be calculated based on the distance from the grid to the BS under the condition of determining the height of the BS and the antenna, $n_{t}^{h}$ = 0, 1, …, $N_{t}^{h}$, $n_{t}^{v}$ = 0, 1, …, $N_{t}^{v}$.

For the convenience of distinction, the above model parameters and channel transmission parameters are represented in Table 1.

### 4.2. Average Communication Rate Analysis

The effectiveness of the VQDRL algorithm under this model will be proven by analyzing the average communication rate with the entire grid and the average communication rate with different grids. All simulations were conducted on a desktop equipped with an Intel Core i7-10700 2.9 GHz CPU(Intel, Santa Clara, CA, USA), with each iteration of $1\times 10^{5}$ time slots taking approximately 12 min in our model.

We divided the coverage area of the nearshore BS communication into grids of size 10 × 10. The parameters of each grid and model value can be represented as follows in Table 2 and Table 3.

**Figure 3:**
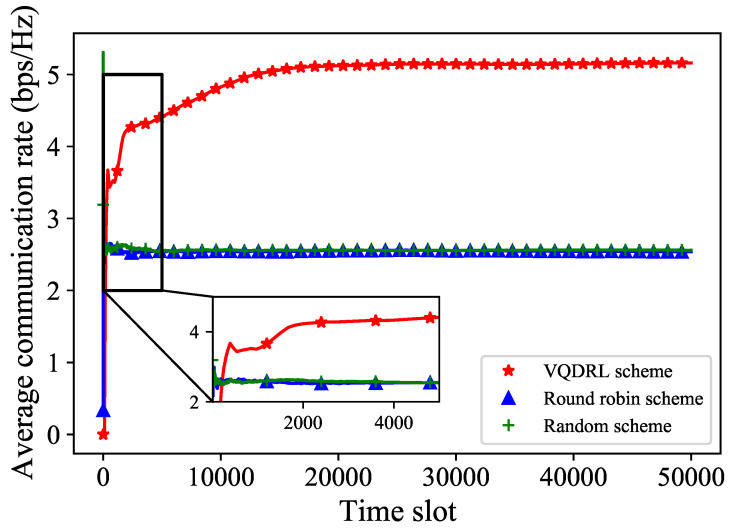
The average communication rate variation of different beam allocation schemes with entire grids.

To observe how the communication rate of the entire maritime environment changes with the communication time slots, we used the average communication rate to show the variation in Figure 3. Here, we employed random beam allocation and round robin beam allocation schemes as comparative simulation results. The scheme that randomly allocates a beam to the current communication grid is called the random beam allocation scheme. Meanwhile, the scheme that allocates a beam to the current grid in ascending order by beam index, and restarts the cycle when the maximum beam index is reached, is called the round robin beam allocation scheme.

It is shown that, as the time slots increase, the difference in average communication rates between the three beam allocation schemes becomes larger. In particular, the round robin beam allocation scheme and random beam allocation scheme do not show a significant difference in the average communication rate for entire maritime grids. Furthermore, with the increase of time slots, the average communication rate for the entire maritime environment under the two schemes essentially remains consistent. Compared to the other two beam allocation schemes, the proposed VQDRL resources allocation scheme significantly improves the average communication rate for the entire maritime environment, and it gradually converges with the increase of the time slot.

**Figure 4:**
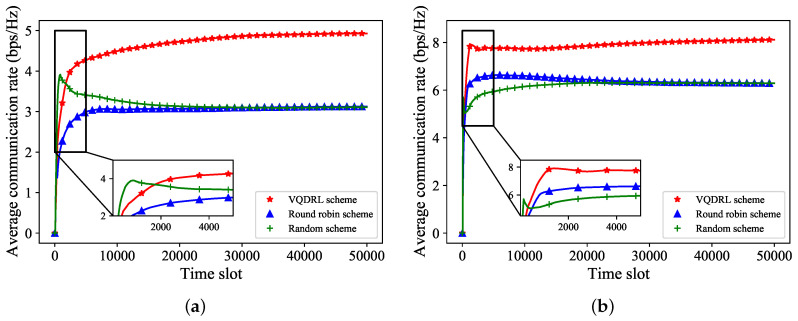
The average communication rate of different beam allocation schemes with different grids. (**a**) ${\mathrm{Grid}}_{55}$. (**b**) ${\mathrm{Grid}}_{95}$.

To further analyze the average communication rate of the maritime environment, we sampled some grids to observe their average communication rate. In Figure 4, we take two grids, ${\mathrm{Grid}}_{55}$ and ${\mathrm{Grid}}_{95}$, as our observation points to observe the average communication rate. The left subplot shows the variation of the average communication rate over time for ${\mathrm{Grid}}_{55}$, while the right subplot shows the variation of the average communication rate over time for ${\mathrm{Grid}}_{95}$. It is shown that, as the time slots increase, the average communication rate of the proposed VQDRL algorithm gradually increases until it stabilizes and converges. Meanwhile, the average communication rates of the two beam schemes in the comparative simulation gradually converge and eventually become consistent as the number of time slots increases. The performance of the random and round robin beam allocation schemes is worse than the performance of the proposed VQDRL algorithm. This trend aligns with the observations in Figure 3.

### 4.3. Awaiting Time per Transmission and Average Virtual Queue Length

**Figure 5:**
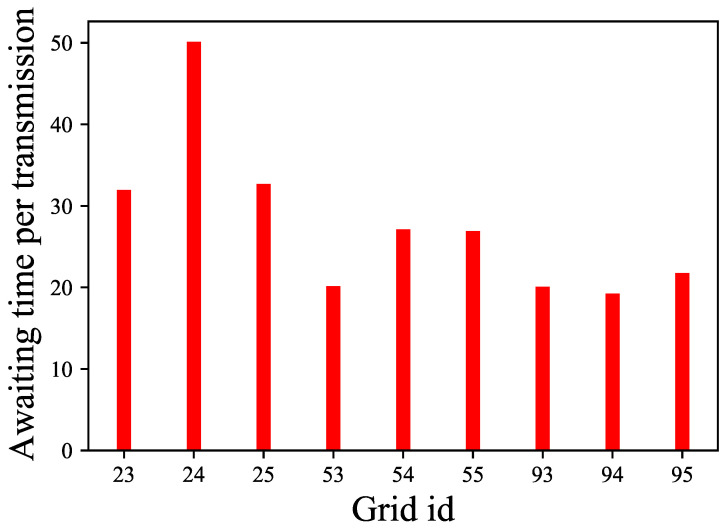
The average waiting time slot of different grids.

In this subsection, we discuss the waiting time per transmission and the average virtual queue length of different grids. Let us set an upper bound on the length of the virtual queue, denoted as $V_{max}$, for all grids. When the virtual queue length of any grid exceeds this value, we consider the grid to be in a communication waiting state. Here, we set $V_{max}=1$, and Figure 5 shows the average waiting time per transmission of different grids over $5\times 10^{4}$ time slots.

**Figure 6:**
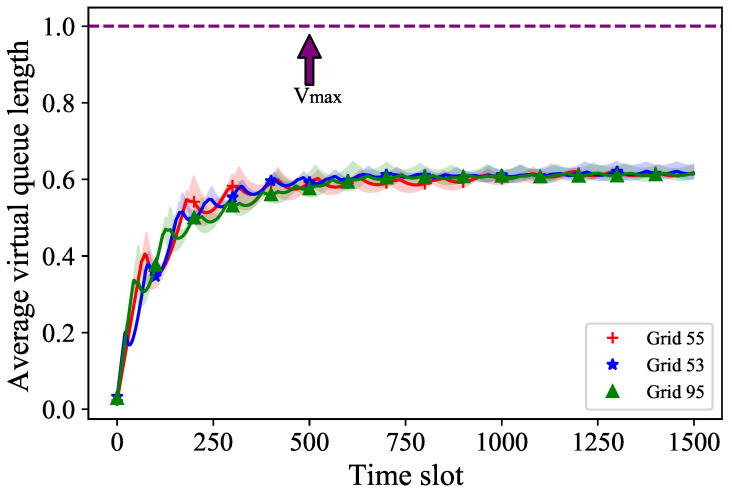
The average virtual queue length over communication time slots.

Meanwhile, we sampled some grids to obtain the average virtual queue length and the confidence interval for the average queue length in Figure 6. The average virtual queue length denotes the growth trend of virtual queues for different grids. It can be calculated by $\frac{\sum_{k=1}^{t}V_{m}\left(k\right)}{t}$. The average virtual queue lengths and their respective confidence intervals under multiple simulations for ${\mathrm{Grid}}_{55}$, ${\mathrm{Grid}}_{53}$, and ${\mathrm{Grid}}_{95}$ are displayed in red, blue, and green, respectively. The purple line represents the upper bound of the virtual queue, and the grids whose virtual queue length exceeds the line at any time slot are considered to be in a waiting state until they are successfully communicated, reducing the virtual queue length to below that line. We observed that the average virtual queue length under these three different grids increases with the growth of time slots and eventually converges to around 0.6, which is often associated with the ability to communicate in each time slot.

### 4.4. Hyperparameters Analysis

In this subsection, we adjust buffer size, learning rate, and batch size to observe the variation of the average communication rate.

**Figure 7:**
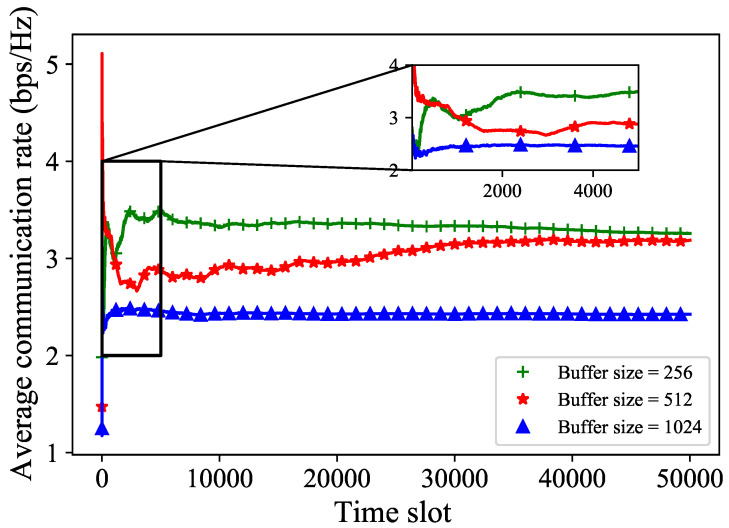
The average communication rate with different buffer sizes.

We adjust the buffer size in Figure 7. At the beginning of the simulation, the average communication rate with a buffer size of 256 is significantly higher than that of the other two batch sizes. As the number of time slots increases, the average communication rate of the buffer size 512 gradually increases and converges near the average communication rate of the buffer size 256. The average communication rate of the largest buffer size remained much lower than the aforementioned two buffer sizes throughout the simulation. This is because when the buffer size is too large, selecting data randomly from replay memory may include some outdated data, which affects the effectiveness of the simulation. In the simulation, we can adjust different buffer sizes to improve the average communication rate in the maritime environment.

**Figure 8:**
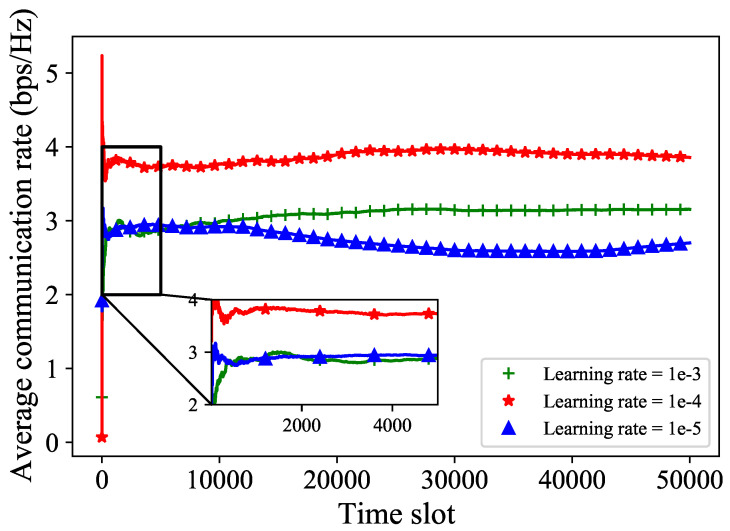
The average communication rate with different learning rates.

As shown in Figure 8, when we set the learning rate to $1\times 10^{-3}$ and $1\times 10^{-5}$, we can see that the convergence curves of the average communication rate are relatively less for both grids. However, when the learning rate is set to $1\times 10^{-4}$, the average communication rate shows the best performance. This is because a learning rate that is too large or too small can cause the beam selection to get stuck in local optima, resulting in poorer performance. We can set a moderate learning rate value to obtain the best performance.

**Figure 9:**
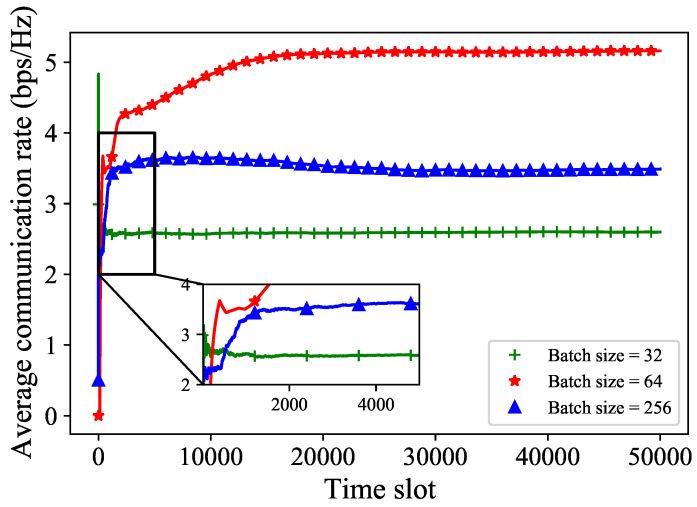
The average communication rate with different batch sizes.

In Figure 9, we adjust the batch size. We can see that when the batch size is 64, the average communication rate performs better compared to the case with a batch size of 32 and 256, as the number of communications increased. This is because, when the batch size is 32, the training data is not sufficient to adequately represent the data features stored in the replay memory, leading to poor performance. And when the batch size is 256, a larger batch size will rely on training data that are too “old” and degrade the convergence performance. Furthermore, a large batch size will consume more time when training neural networks. Therefore, an appropriate batch size plays a crucial role in training neural networks.

## 5. Conclusions

In this paper, a VQDRL resources allocation scheme was proposed and investigated in the maritime environment, and we discussed the average communication rate performance. Firstly, a maritime communication model with grid-based partitioning was employed, and we utilized VQDQN for allocating beam resources. Secondly, the average communication rate of all grids and the average communication rates of different grids were simulated and the simulation results demonstrate the effectiveness of our beam allocation scheme. Additionally, we discussed the performance of average waiting per transmission, average virtual queue length, and confidence intervals of different grids. Finally, we adjusted hyperparameters to obtain a better performance on the average communication rate. The simulation results demonstrated that we can construct suitable beam allocation schemes to maximize the communication rate in the maritime environment using our algorithm. Furthermore, further work can be conducted by adopting the objective function of our proposed scheme to analyze various network performance metrics, including energy efficiency and spectrum utilization. Moreover, improving the generalization performance of our proposed scheme requires further investigation.

## Figures and Tables

**Table 1:** Summary of Key Notations.

Notations	Meaning
M;N	Set of grids and beam resources
$\theta_{m};\phi_{m}$	Horizontal and vertical angle from grid *m* to BS
$y_{m,n}$	Receive signal from beam *n* to grid *m*
$x_{m}$	Transmit signal to grid *m*
$P_{t};\alpha$	Transmit power and path loss coefficient
$G_{m}$	Antenna gain for grid *m*
$n_{0}$	Additive complex white Gaussian noise
h	Channel transfer matrix
w	Unit-form precoding vector
S;A	Set of state information and available action
r(t)	Reward function
$R_{m,n}$	Communication rate of grid *m* with beam *n*
$Q\left(\theta \right);Q\left(\theta^{-}\right)$	Q-network and target network
$r_{m}$	Requirements of maritime users within grid *m*
$p_{m}$	Communication requirement of grid *m*
$V_{m};K$	Virtual queue length of grid *m* and rice factor

**Table 2:** Grid parameters.

$s_{0}\{5$ km, $\frac{1}{6}\pi \}$	...	$s_{4}\{5$ km, 0}	...	$s_{9}\{5$ km, $-\frac{1}{6}\pi \}$
*…*	*…*	*…*	*…*	*…*
$s_{50}\{2.5$ km, $\frac{1}{6}\pi \}$	*…*	$s_{54}\{2.5$ km, 0}	*…*	$s_{59}\{2.5$ km, $-\frac{1}{6}\pi \}$
*…*	*…*	*…*	*…*	*…*
$s_{90}\{0.5$ km, $\frac{1}{6}\pi \}$	...	$s_{94}\{0.5$ km, 0}	*…*	$s_{99}\{0.5$ km, $-\frac{1}{6}\pi \}$

**Table 3:** Simulation parameters and values.

Parameters	Values
$N_{t}^{h};N_{t}^{h}$	$N_{t}^{h}=8;N_{t}^{h}=8$
$N_{h};N_{v}$	$N_{h}=4;N_{v}=4$
M;N	M=100;N=16
$P_{t};G_{m}$	$P_{t}=1000;G_{m}=1$
α;β	α=1;β=0.001
ϵ;γ	ϵ=0.9;γ=0.99
$\xi ;R_{th}$	$\xi =4;R_{th}=1$
buffer size; batch size	buffer size = 512; batch size = 64
learning rate; *K*	learning rate = $1\times 10^{-4}$; K=9
$N_{t};\eta$	$N_{t}=20;\eta =10$

## Data Availability

The data presented in this study are available on request from the corresponding author.
